# Subterranean, Herbivore-Induced Plant Volatile Increases Biological Control Activity of Multiple Beneficial Nematode Species in Distinct Habitats

**DOI:** 10.1371/journal.pone.0038146

**Published:** 2012-06-27

**Authors:** Jared G. Ali, Hans T. Alborn, Raquel Campos-Herrera, Fatma Kaplan, Larry W. Duncan, Cesar Rodriguez-Saona, Albrecht M. Koppenhöfer, Lukasz L. Stelinski

**Affiliations:** 1 Entomology and Nematology Department, Citrus Research and Education Center, University of Florida, Lake Alfred, Florida, United States of America; 2 Center for Medical, Agricultural, and Veterinary Entomology, Agricultural Research Service, U.S. Department of Agriculture, Gainesville, Florida, United States of America; 3 Departamento de Contaminación Ambiental, Instituto de Ciencias Agrarias, Centro de Ciencias Medioambientales, Madrid, Spain; 4 Department of Entomology, Rutgers University, New Brunswick, New Jersey, United States of America; Centro de Investigación y de Estudios Avanzados, Mexico

## Abstract

While the role of herbivore-induced volatiles in plant-herbivore-natural enemy interactions is well documented aboveground, new evidence suggests that belowground volatile emissions can protect plants by attracting entomopathogenic nematodes (EPNs). However, due to methodological limitations, no study has previously detected belowground herbivore-induced volatiles in the field or quantified their impact on attraction of diverse EPN species. Here we show how a belowground herbivore-induced volatile can enhance mortality of agriculturally significant root pests. First, in real time, we identified pregeijerene (1,5-dimethylcyclodeca-1,5,7-triene) from citrus roots 9–12 hours after initiation of larval *Diaprepes abbreviatus* feeding. This compound was also detected in the root zone of mature citrus trees in the field. Application of collected volatiles from weevil-damaged citrus roots attracted native EPNs and increased mortality of beetle larvae (*D. abbreviatus*) compared to controls in a citrus orchard. In addition, field applications of isolated pregeijerene caused similar results. Quantitative real-time PCR revealed that pregeijerene increased pest mortality by attracting four species of naturally occurring EPNs in the field. Finally, we tested the generality of this root-zone signal by application of pregeijerene in blueberry fields; mortality of larvae (*Galleria mellonella* and *Anomala orientalis*) again increased by attracting naturally occurring populations of an EPN. Thus, this specific belowground signal attracts natural enemies of widespread root pests in distinct agricultural systems and may have broad potential in biological control of root pests.

## Introduction

Natural enemies of herbivorous pests use flexible foraging strategies that often incorporate environmental cues emitted by the herbivore’s host plant. While the role of herbivore-induced volatiles in plant-herbivore-natural enemy interactions is well-documented aboveground [1]–[5], new evidence from several systems, including strawberry, maize and, most recently, citrus, indicates that induced root volatiles may protect plants by attracting entomopathogenic nematodes (EPNs) [6]–[12]. However, to date, only one root-induced attractant has been described and shown to enhance the effectiveness of EPNs in the field: (*E*)-β-caryophyllene from the roots of maize (*Zea Mays* L.) [11], [13]. The disparity in the number of aboveground investigations versus analogous belowground research on indirect defense is largely due to technical limitations rather than a lack of ecological or agricultural relevance [9], [14]. No previous studies have detected a belowground herbivore-induced volatile from intact plants in the field or measured the effectiveness of belowground attractants for recruiting populations of naturally occurring EPNs in the soil. Depending on the specificity of interactions, the identification and manipulation of a root signal in the field could well enhance biological control of diverse root pests in agroecosystems.

Larvae of the weevil *Diaprepes abbreviatus* (L.), introduced into Florida in 1964 [15], feed on the roots of more than 290 plant species including citrus, sugarcane, potatoes, strawberries, sweet potatoes, papaya, and non-cultivated wild plants [16]. Over the past 40 years, the weevil has significantly contributed to the damage and spread of disease in agricultural plants [17]. Because pesticides are expensive, environmentally hazardous and often ineffective [18], [19], currently the most effective alternative method of root-pest control is the application of EPNs from the genera *Heterorhabditis* and *Steinernema* [20]. EPNs are obligate parasites that kill their host with the aid of a symbiotic bacterium [21], [22]. Over its 20 years of use, the efficacy of mass release of EPNs as a biopesticide for *D. abbreviatus* has been reported as varying and unpredictable, ranging anywhere between 0 to >90% [23]. Promoting plant attractiveness to natural enemies is a novel alternative to traditional broad-spectrum pesticides, which indiscriminately kill predators and parasitoids and often lead to subsequent pest resurgence [24]–[27]. Deploying herbivore-induced plant volatiles (HIPVs) aboveground by controlled release dispensers has been shown to increase recruitment and retention of beneficial natural enemies to plants [27]–[29]. In an analogous belowground investigation, EPN infection of western corn rootworm (*Diabrotica virgifera virgifera* LeConte) larvae was increased by spiking soil surrounding maize roots with the HIPV, (*E*)-β-caryophyllene [11]. Herein, we investigated the mechanisms by which a novel HIPV affects naturally occurring EPN species and their consequence on belowground herbivores in two distinct agroecosystems.

We have recently shown that a citrus root stock (*Citrus paradisi* Macf. × *Poncirus trifoliata* L. Raf.) releases HIPVs in response to larval feeding by the weevil, *D. abbreviatus*, and that these HIPVs attract EPN species in lab bioassays [7], [8]. Here we characterize the specific HIPV attractant as 1,5-dimethylcyclodeca-1,5,7-triene (pregeijerene) and show its real-time release in response to herbivory. We also demonstrate that field application of this volatile increases mortality of belowground root weevils by attracting naturally occurring nematodes. We used recently developed qPCR primers and probes to detect and enumerate cryptic species of EPNs allowing for species-specific quantification of nematode response to attractants belowground. Given the broad effect of pregeijerene on EPN species, we also tested and demonstrated its efficacy in a non-citrus, temperate climate agroecosystem. The use of plant-produced signals, such as the damage-induced release of pregeijerene, along with conservation biological control strategies, could extend the usefulness of EPNs in crops damaged by belowground herbivores.

## Results

### Induction of Root Volatiles

**Figure 1:**
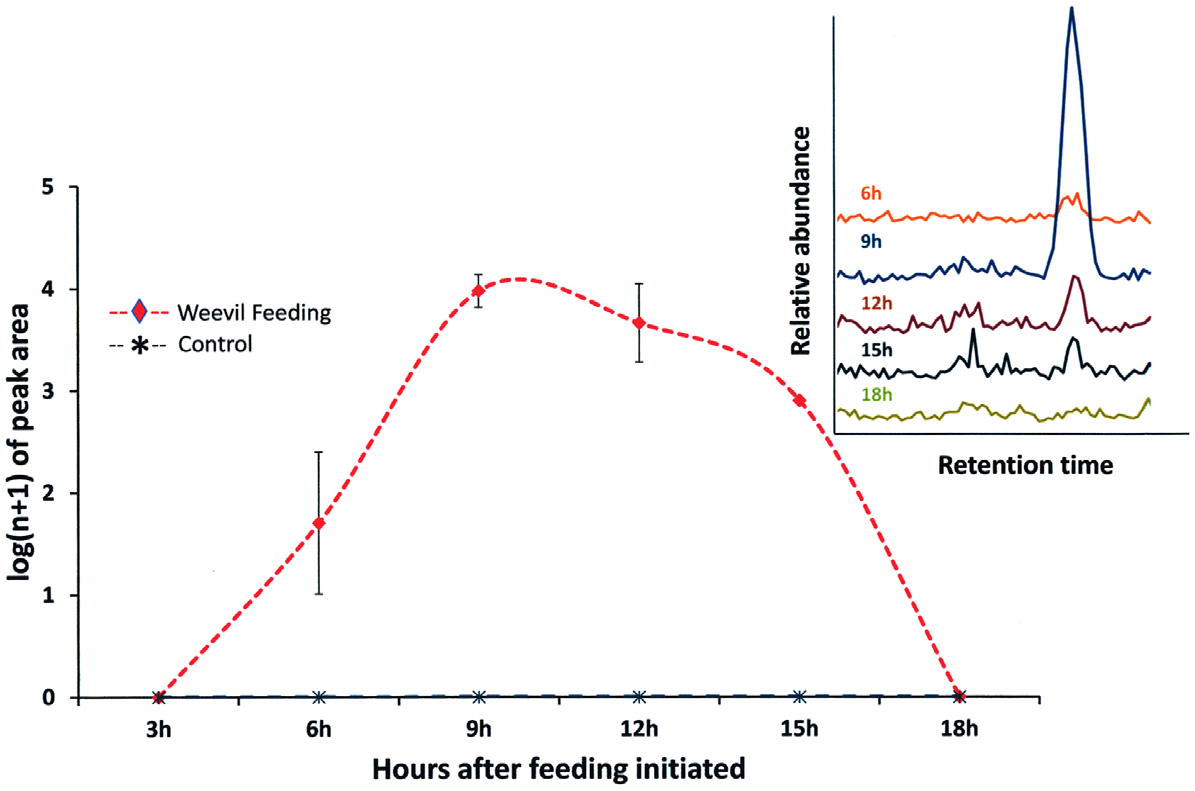
Time course of pregeijerene (1,5-dimethylcyclodeca-1,5,7-triene) release following initiation of weevil (*Diaprepes abbreviatus*) feeding on citrus roots. Insert in the upper right displays chromatogram of volatile abundance at each interval.

Volatiles were non-destructively sampled every three h from the root zone of citrus seedlings in glass chambers (Analytical Research Systems, Gainesville, FL) with sandy soil [8]. Gas chromatography-mass spectrometry (GC-MS) revealed 1,5-dimethylcyclodeca-1,5,7-triene (pregeijerene) as the dominating volatile, reaching a maximum release between nine and 12 h after initiation of larval feeding (Figure 1). There was an effect of treatment (*F*_1,4_ = 26.4, *P = *0.0005) and volatile release over time (*F*_3,2_ =  2812, *P* = 0.0005). A stainless steel probe (Figure S3) was designed to collect volatiles in the field from the soil beds surrounding citrus trees in an unmanaged orchard. Here, GC-MS analyses again revealed pregeijerene in the root zone, as the most abundant volatile at one m away from the trunks of trees and still at detectable levels at 10 m from trees (Figure S4).

### Nematode Attraction in the Field using HIPVs

We next conducted field tests to determine whether application of volatiles collected from infested roots would impact EPN-inflicted mortality of sentinel *D. abbreviatus* larvae. Commercially available EPN had been applied to the test orchard at numerous occasions; however, their persistence was not monitored. Cylindrical mesh cages containing a single *D. abbreviatus* larva in autoclaved sandy soil [30] were treated with: (i) volatiles collected from weevil-infested roots or (ii) a blank solvent control (Figure S5). Larval mortality was 74±6.9% in the presence of volatiles from infested roots, but only 41±7.5% in the solvent alone treatment (N = 10, *t_18_* = 2.75, *P* = 0.013).

### Nematode Attraction in the Field using Isolated Pregeijerene

**Figure 2:**
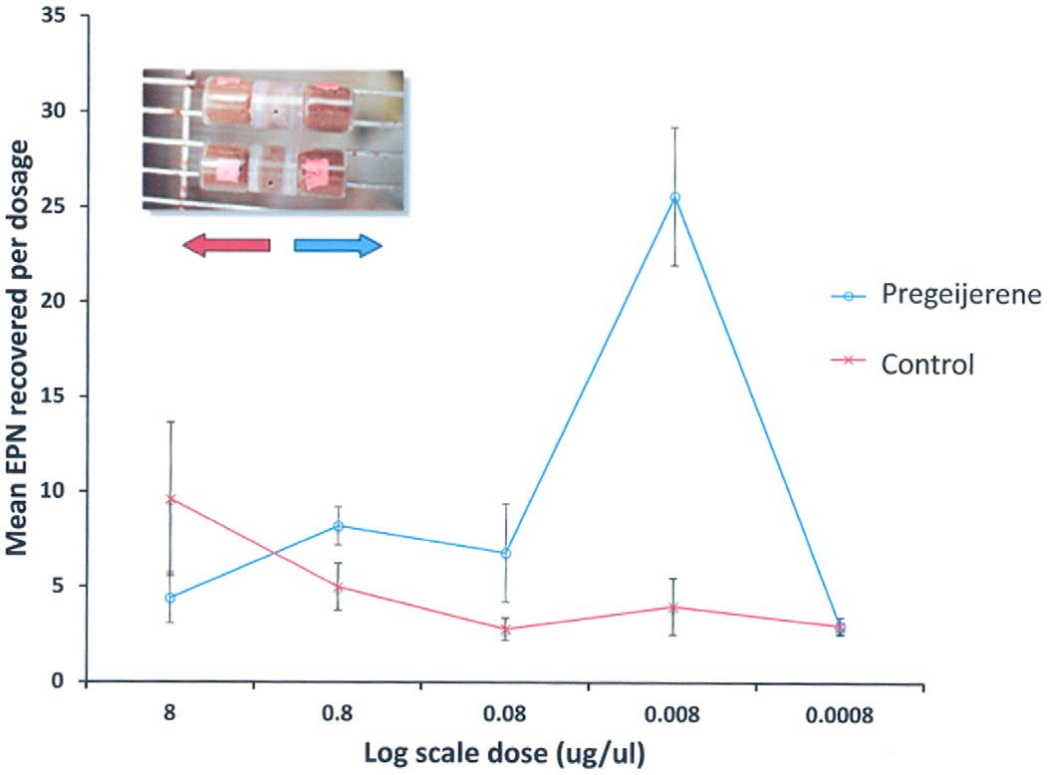
Optimal dosage of pregeijerene (1,5-dimethylcyclodeca-1,5,7-triene) for attracting entomopathogenic nematodes (*Steinernema riobrave* and *Heterorhabditis indica*) based on the log scale dilution of purified compound. Picture in upper left displays sand-filled two-choice olfactometers used for nematode bioassays.

**Figure 3:**
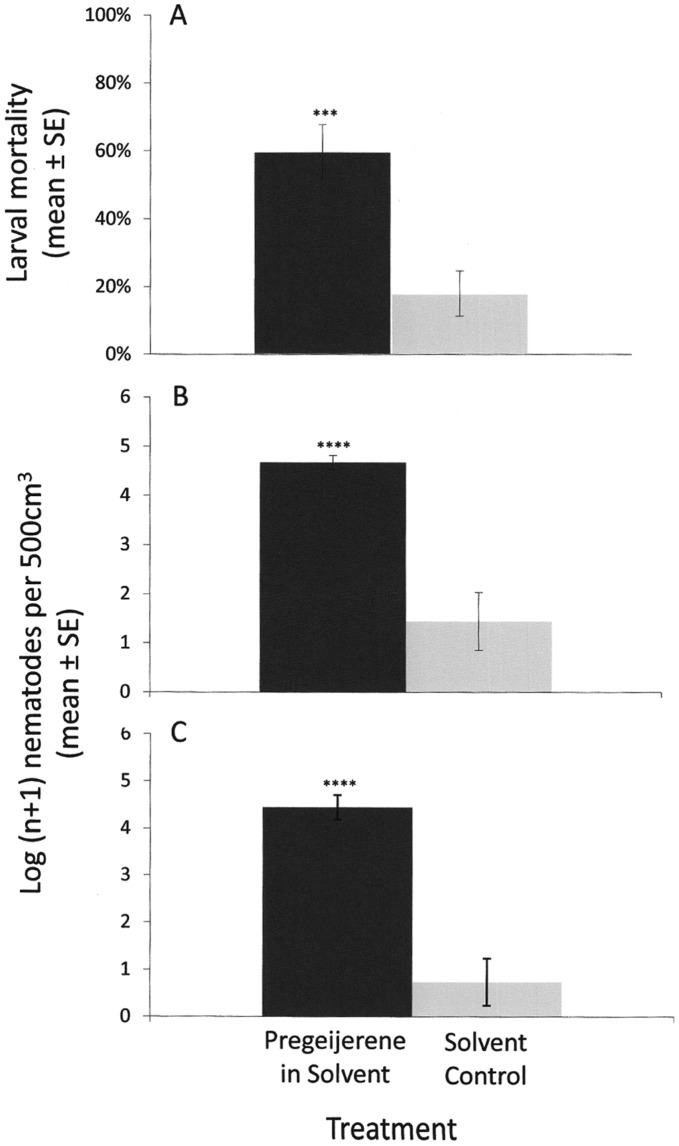
Effect of pregeijerene on mortality of *Diaprepes abbreviatus* larvae and associated attraction of entomopathogenic nematode infective juveniles (IJs) (all species combined). A) Average mortality of larvae buried with purified pregeijerene compared with the solvent control (N = 10, *t = *4.01, *P = 0.0008*). B) Mean number of IJs recovered from cages containing purified pregeijerene compared with cages containing the solvent control (N = 10, *t = *5.33, *P = 0.00005*). C) Mean number of IJs recovered from soil samples surrounding cages containing pregeijerene compared with the solvent control (N = 10, *t = *5.67, *P = 0.00003*).

A second experiment tested whether pregeijerene alone would increase mortality of larvae by attracting EPNs. For these experiments, a sufficient amount of pregeijerene was first extracted and purified from the roots of Common Rue (*Ruta graveolens* L.). The structure of extracted pregeijerene was confirmed by nuclear magnetic resonance (NMR) (Table S1, Table S2). To test for the attractiveness of pregeijerene, we first used serial dilutions of purified compound in dichloromethane in two-choice, sand-filled olfactometers [7], [8], [31]; 8 ng/µl (in 30 µl aliquots) was found to be the optimally attractive dosage to EPNs (*S. riobrave* and *H. indica*) (Figure 2). We used real-time qPCR to quantify the attraction of naturally occurring EPNs in the field and identified them to species. Our approach was to use species-specific primers and probes to identify EPN species known to either naturally occur in Florida: *S. diaprepesi, H. indica*, *H. zealandica*, and *Steinernema* sp. LWD1 (an undescribed species in the *S. glaseri* group); those which were applied to citrus orchards in the form of commercial biopesticides (*S. riobrave*); or those which might be introduced from natural long-distance spread from pastures and golf courses to manage mole crickets (*S. scapterisci*) [32], [33]. Mortality of larvae buried with purified pregeijerene was > 3-fold higher than that of larvae buried with the solvent control (Figure 3A). The number of EPNs detected within (Figure 3B) and around (Figure 3C) cages containing purified pregeijerene was significantly higher than that from cages with the solvent control. Tukey HSD test indicated *H. indica* and *H. zealandica* were more abundant than *Steinernema* sp. LWD1 and *S. diaprepesi* (*P* < 0.0001 in all comparisons); however, there were no differences in the relative representation of species between treated and control samples. Neither *S. riobrave*, nor *S. scapterisci* were detected in any of the samples.

### Manipulation of Nematode Behavior with Pregeijerene in Blueberry Plantings

Finally, we tested the generality of pregeijerene as an EPN attractant by deploying the compound in a geographically distant, non-citrus agricultural system: commercial highbush blueberry, *Vaccinium corymbosum* L., in Chatsworth, NJ. No pregeijerene was detected in volatiles collected from soil surrounding blueberry roots. Cages (described above) containing either a third-instar oriental beetle, *Anomala orientalis* Waterhouse, a scarab blueberry root pest or a late instar greater wax moth, *Galleria mellonella* L., larva (a widely used EPN sentinel), were deployed in blueberry fields. As described above, cages were treated with either blank solvent or pregeijerene. EPN-inflicted larval mortality (combined *A. orientalis* and *G. mellonella*) was nearly 2-fold greater in treatments with pregeijerene (55%) than those with solvent alone (30%) (*P* = 0.009). The increase was highly significant (from 40% to 80%) for *G. mellonella* (*P* = 0.003), but not statistically significant (from 20% to 30%) for *A. orientalis* (*P = *0.552). Emerging EPNs were identified as *S. glaseri* with real-time PCR. On average, there were more *S. glaseri* nematodes surrounding the treatment (Mean±SE, 7.96±2.91) than in the control (4.43±2.56); however, this difference was not significant (N = 10, *t_18_ = 0.909, P = *0.38).

## Discussion

The obstacles of investigating belowground chemically mediated interactions between plants and animals are being overcome gradually, opening opportunities for manipulating these interactions for enhanced biological control [34]–[36]. At least half of all plant biomass is attacked by underground herbivores and pathogens, living in a complex ecological food web in the soil [37]. Although induced plant responses were originally postulated as a potential novel approach to pest management in agricultural systems [38] for insect herbivore population regulation [39], few studies [40]–[43] of induced responses (particularly volatiles) have addressed their practical application beyond fundamental concepts in ecology and evolutionary biology [35], [37], [43], with particularly few studies for belowground systems. HIPVs are likely important mediators of tritrophic interactions that afford indirect plant defense within the root zone. Our study not only shows this approach in the field, but also provides the first description of an ecological role for the C_12_ terpene, pregeijerene.

To evaluate applied volatiles for the attraction of belowground natural enemies in the field, studies usually quantify mortality of a target pest by trapping adults emerging from soil [11]. This technique frequently results in low recovery and also gives no confirmation of the specific cause of mortality. In addition, it can be difficult to quantify populations of naturally occurring EPNs, which may be abundant in soil, but remain cryptic. We used real-time qPCR as an efficient method for describing EPN diversity and quantifying their abundance [32], [44]–[46]. Moreover, we showed that pregeijerene was directly responsible for attracting five species of native EPNs in the soil so as to enhance pest mortality. Given the efficacy of this compound, there may be little need for exogenous application of non-native EPNs in systems with a rich fauna of native EPNs. In orchards with established EPN populations, large-scale introduction of non-native species may temporarily reduce native populations due to trophic cascades that increase predatory fungi and attenuate net efficacy of biological control [19], [47]. Although it is known that artificially reared and commercially formulated EPNs can persist, it is possible that natives have advantages associated with habitat acclimation and response to HIPVs [9]; thus, further investigation of enhancing conservation biological control of belowground pests in concert with behavioral modification via HIPVs is warranted.

The results of the experiment conducted in blueberries, an agricultural setting vastly different from citrus, demonstrate the potential broad applicability of pregeijerene on diverse species of EPNs. Timing application of pregeijerene to target the most susceptible instar of *A. orientalis* should optimize its efficacy (depending on EPN species, final (third)-instar *A. orientalis* may be less susceptible to EPN infection than earlier larval instars [48]).

Our previous research suggests that volatile production in response to herbivore feeding differs between citrus species [8]. Thus, our current findings could have broad impacts not only for rootstock selection in commercial citriculture, but also for use of attractants in other agroecosystems as demonstrated in blueberry fields. Here, we identified an additional naturally occurring species of EPN responsive to pregeijerene that was not found in Florida. Pregeijerene may thus have extensive application for enhancing native biological control of root feeding insects, including those, which attack a wide range of crops. However, we recognize that plants should benefit from the proposed function of herbivore-induced responses. Our experiments were not designed to assess improved crop fitness as a result of attracting beneficial natural enemies by application of pregeijerene. However, there is strong evidence that plants benefit from the cascading effects caused by EPN-induced suppression of herbivores [49], and our future work will determine whether crop yield is affected by HIPV-mediated manipulation of EPN behavior.

Where most aboveground studies have identified blends of volatiles as being responsible for the attraction of natural enemies [50], analogous belowground studies that identify an attractant in an agricultural system have demonstrated that a single compound can elicit natural enemy responses. This certainly makes application potentially less complex, but also points to an interesting potential property of belowground cues and natural enemy response. Future work should evaluate the complexity of belowground cues and the range of volatiles that cause belowground natural enemies, like EPNs, to respond. Only recently have new methodologies been employed to investigate chemicals stimulating changes in EPN behavior [51] and much more progress is necessary to understand these relationships [52].

## Materials and Methods

### Insect Larvae

*D. abbreviatus* larvae were obtained from a culture maintained at University of Florida’s Citrus Research and Education Center (CREC) in Lake Alfred, FL. This culture was periodically supplemented from a larger culture maintained at the Division of Plant Industry Sterile Fly Facility in Gainesville, FL. Larvae were reared on a commercially prepared diet (Bio-Serv, Inc., Frenchtown, NJ) using procedures described by Lapointe and Shapiro [53]. Larvae used in experiments were from third to sixth instars.

Third-instar *A. orientalis* were collected from untreated turf areas at the Rutgers University Horticultural Research Farm (North Brunswick, NJ) in late April. The larvae were stored individually in the cells of 24-well plates in sandy loam at 10°C for two weeks and returned to room temperatures (21–24°C) for 24 h before use in experiments. Late instar *G. mellonella* larvae were obtained from Big Apple Herpetological (Hauppauge, NY).

### Plants

‘Swingle citrumelo’ (*C. paradisi* Macf. × *P. trifoliata* L. Raf.) rootstock is very prominent in commercial citrus production [54]. The extensive use of this rootstock in commercial citrus production justified its use in this investigation. All plants were grown and maintained at the CREC in Lake Alfred, FL in a greenhouse at 26±3°C and 60–80% RH. Citrus seedlings used in the experiments were 25–35 cm long. *R. graveolens* was purchased as full grown plants 46–61 cm in height. The plants were immediately bare rooted and rinsed to remove as much soil material as possible; only roots were placed into vials containing dichloromethane for further extractions and purification.

### Nematodes Used for Laboratory qPCR and Bioassays

The entomopathogenic nematodes, *S. diaprepesi* HK31, *S. riobrave* Btw1, *Steinernema sp.* LWD1, *H. indica* Ker1 and *H. zealandica* Btw1 were isolated from *D. abbreviatus* larvae buried in commercial citrus orchards in Florida. *S. riobrave* and *S. carpocapsae* isolates were descendants of commercial formulations intended for field application to manage *D. abbreviatus*. Other EPN species included in this study were *S. scapterisci* (provided by Dr. J.H. Frank, University of Florida, FL). All EPN species were cultured in last instar larvae of the greater wax moth, *G. mellonella larvae*, at approximately 25°C according to procedures described in Kaya and Stock [55]. Infective juveniles (IJs) that emerged from insect cadavers into emergence traps were stored in shallow water in tissue culture flasks at 15°C for up to two weeks prior to use.

### *In situ* Volatile Collection from Infested Roots in the Greenhouse

Six ‘Swingle citrumelo’ plants were initially placed in glass root-zone chambers (300 ml volume capacity) (Analytical Research Systems, Gainesville, FL) filled with sand that had been autoclaved for one hour at 121°C and then adjusted to 10% moisture as described in Ali et al. [7], [8] and El-Borai et al. [31]. All seedlings were given three days to adjust to their sand filled chambers. Three of the plants were subjected to feeding by weevil larvae for three days; the remaining three served as undamaged controls. During this period, each of the six root-zone chambers were connected to a vacuum pump (Analytical Research Systems, Gainesville, FL) with a suction flow of 80 ml/min [7]. Compounds emitted from chambers were collected on adsorbent traps filled with 50 mg Super-Q (800–1000 mesh, Alltech, Deerfield, IL) held in glass fittings between the chamber and vacuum pump [7]. Super-Q traps were replaced every three h for a 72-h period to track the time course of volatile release. The removed Super-Q traps were subsequently eluted with 150 µl of dichloromethane into individual 2.0 ml clear glass vials (Varian, Part Number 392611549, equipped with 500 µl glass inserts) [7]. The amount of pregeijerene detected over time for all treatments was subjected to multiple analysis of variance (MANOVA) to determine differences between, as well as, within treatments.

It was a challenge to remove sufficient pregeijerene from infested roots for bioassays and field testing. However, it was previously established [56] that a hydrodistillate of Common Rue (*Ruta graveolens*) roots contained the related terpene, geijerene, as a major constituent (67% of the total volatile compounds). Pregeijerene easily converts to geijerene at temperatures exceeding 120°C [57] (Figure S1). On-column GC-MS analyses confirmed pregeijerene as the main naturally occurring terpene in roots of Common Rue that could be easily extracted and purified from crushed roots using a series of solid phase extractions (Figure S2).

### *In situ* Volatile Collection from Infested Roots in the Field

Volatiles were collected from the soil beds surrounding citrus trees in the field. A soil probe (Figure S3) was used to sample soil volatiles at a depth of 20 cm and at distances of one and 10 m from the trunks of citrus trees. A vacuum pump was used to pull air at a rate of 200 ml/min for a total of 30 min. Compounds were collected on adsorbent traps filled with 50 mg of Super-Q attached to the top of the soil probe (Figure S3). The Super-Q traps were subsequently eluted as described in the previous section.

### Identification of Pregeijerene

Pregeijerene isolated from Common Rue and that from citrus roots after herbivore feeding was identified by electron impact (EI) and chemical ionization (CI) GC-MS analyses on DB1, DB5, and DB35 GC columns. Although the EI mass spectra matched pregeijerene in the Adams 2 library, the lack of a standard made it necessary to confirm the structure by nuclear magnetic resonance (NMR) (described in Supporting Information Materials and Methods S1).

### Two-Choice Bioassay to Determine Optimal Dosage to Attract EPNs

The behavioral responses of EPNs to collected pregeijerene were quantified in a two-choice, sand-filled olfactometer [7], [31]. Briefly, the olfactometer consists of three detachable sections: two opposing 16-ml glass jars which contained treatments and a central connecting tube three cm in length with an apical hole into which EPN were applied. Dilutions from the purified *R. graveolens* root extract were placed on filter paper, which was allowed to dry for 30 s to allow solvent evaporation. Thereafter, filter papers were placed on the bottoms of each glass jar, which were then filled with moist (10% w/v) sterilized sand. The central chamber connecting the two arms of the olfactometer was also filled with sterilized and moistened sand. EPNs (*ca.* 200 IJs) were applied into the central orifice of the connecting tube and given eight h to respond. Following the incubation period, the column was disassembled and the IJs from the two collection jars were extracted using Baermann funnels. The experiment was replicated five times for each dilution and separately tested with two EPN species: *S. riobrave* Btw1 and *H. indica* Ker1.

A student’s *t*-test was used to compare nematode response in the two-choice olfactometer. Since responses of both species to pregeijerene *versus* the solvent controls were identical, data for both species were combined prior to analysis (*df = *18). The dosage at which a significant proportion of EPNs were attracted to the treatment arm was selected for our field trial.

### Application of HIPVs in the Field

An experiment was conducted in a sandy soil (97∶2:1, sand:silt:clay; pH 7.1; 0.1% OM) citrus orchard at the CREC (28 07 26.84 N, 81 42 55.31 W). The experiment was placed within a section of mature orange trees spaced (without beds) 4.5 m within and 8.1 m between rows that was irrigated with microsprinklers. A randomized design was used to place treatments between trees in eight adjacent rows. Cylindrical wire-mesh cages containing autoclaved sandy soil (10% moisture) and a single *D. abbreviatus* larva (reared on artificial diet for 3 to 5 weeks) were buried 20 cm deep in the soil beneath the tree canopies. Cages were made of 225-mesh stainless-steel cylinders (7 length × 3 cm diam) secured at each end with polypropylene snap-on caps. A replicate consisted of six cages placed equidistantly from one another in a circle pattern (48 cm diam) for each treatment. All cages contained a single *D. abbreviatus* larva and were baited with one of two treatments per replicate: (i) volatiles from roots fed upon by a *D. abbreviatus* larva or (ii) blank solvent control. There were 10 replicates of six cages per treatment. Treatments were applied as 30 µl aliquots to three cm diameter filter paper discs (Whatman). Solvent was allowed to evaporate for 30 s prior to insertion of filter papers at the base of each cage. The cages were left buried for 72 h. Eight soil core samples (2.5 cm diam × 30 cm deep) were taken from soil surrounding the treatment arena before the cages were removed to measure the number of EPNs attracted to the surrounding treatment arena. Recovered larvae were rinsed and placed on moistened filter paper within individual Petri dishes to confirm EPN infection by subsequent infective juvenile emergence from cadavers. Mortality of the larvae caused by EPNs was recorded from 0 to 72 h after removal from soil.

The effect of isolated pregeijerene on larval mortality was investigated in two additional experiments (one of which was conducted in a blueberry planting in Chatsworth, NJ, using *A. oientalis* and *G. mellonella*). The methods for these experiments were similar to those described above, except that the soil remaining within the six cages from each replication was placed in a container and homogenized for later nematode DNA extraction (n = 10). Soil cores taken from the surrounding treatment arenas were also combined and stored for nematode DNA extraction (n = 10). Fisher’s exact test was used to compare larval mortality between the treatment and control. Only soil samples from the citrus experiment were analyzed for DNA quantification.

### Detection, Identification, and Quantification of Entomopathogenic Nematodes Using Real-Time qPCR

Real-time qPCR was used to quantify attraction of naturally occurring EPN species to volatiles applied in the field and to identify nematodes to species. This technique targeted 11 EPN species [32], [33], [44]. In the citrus experiment, we surveyed the natural occurrence of six species (*S. diaprepesi, Steinernema* sp. *glaseri* group, *S. riobrave, S. scapterisci, H. indica,* and *H. zealandica*); in the blueberry experiment, nine species were surveyed (*S. carpocapsae, S. feltiae, S. glaseri, S. kraussei, S. scapterisci, Steinernema* sp. *glaseri* group, *H. indica, H. zealandica,* and *H. bacteriophora*). Briefly, species-specific primers and TaqMan® probes were designed from the ITS rDNA region using sequences of the target species as well as closely related species recovered from the NCBI database or generated by the authors in that study. Multiple alignments of the corresponding sequences were performed [58] to select areas of variability in the ITS region. The designed primers and probes provided no non-specific amplification when they were tested using other EPN species. Standard curve points were obtained from DNA dilution. Four independent DNA extractions were performed from Eppendorf tubes containing 300 IJs in 100 µl of the corresponding nematode species (Ultra Clean SoilTM DNA kit, MO BIO) to generate a standard curve [32], [44]. Dilutions corresponding to 100, 30, 10, 3, and 1 IJs were prepared using serial dilution of the appropriate DNA.

Nematodes from soil samples were extracted by sucrose centrifugation [59] from aliquots of 500 cm^3^ from the mixed composite sample. Each nematode community was concentrated in a 1.5 ml Eppendorf tube. DNA was processed using the UltraCleanTM soil DNA extraction kit and quantification was performed for each DNA extraction using the nanodrop system with the control program (ND-1000 v3.3.0). All DNA samples were adjusted to 0.2 ng/µl that is required for nematode quantification [32]. The resulting real values were analyzed with analysis of variance (ANOVA) for the EPN species recovered (*F = *41, *df = *5, 204). Where ANOVA showed significant differences, Tukey’s HSD test (*P* < 0.05) was conducted to separate means in the software R (R Development Core Team 2004).

## Supporting Information

Figure S1 Conversion of pregeijerene (A) to geijerene (B).
(TIFF)

Figure S2 Chromatograms showing the initial crude extract prior to purification and final purified pregeijerene. The Y-axis represents relative abundance or the ratio of a mass peak profile area to that of another. These were calculated as ratios of the sums of areas used to plot the profiles ×100%.
(TIFF)

Figure S3 Soil probe design used to sample volatiles belowground. Probe is inserted into soil and connected to a vacuum pump.
(TIFF)

Figure S4 Chromatograms of volatiles taken from intact citrus roots in the field at one and 10 m distances from the trunk of the tree. The Y-axis represents relative abundance or the ratio of a mass peak profile area to that of another. These were calculated as ratios of the sums of areas used to plot the profiles ×100%.
(TIFF)

Figure S5 Schematic diagram of the deployment and sampling procedure for field experiments in which sentinel traps with root weevils were deployed with or without HIPVs. One treatment replicate is depicted.
(TIFF)

Table S1 ^1^H (600 MHz), ^13^C (151 MHz), HMBC and NOESY NMR spectroscopic data for pregeijerene in C_6_D_6_.^13^C was also detected directly (126 MHz) using a 5 mm Cryoprobe. Chemical shifts referenced to residual proton signal in C_6_D_6_ benzene δ(^1^H) = 7.16 ppm for ^1^H and δ(C_6_D_6_H) = 128.2 ppm for ^13^C.
(DOCX)

Table S2 ^1^H (600 MHz), ^13^C (151 MHz), HMBC and NOESY NMR spectroscopic data for geijerene in C_6_D_6_.^13^C was also detected directly (126 MHz) using a 5 mm Cryoprobe. Chemical shifts referenced to residual proton signal in C_6_D_6_ benzene δ(^1^H) = 7.16 ppm for ^1^H and δ(C_6_D_6_H) = 128.2 ppm for ^13^C. For convenience, the pregeijerene numbering is retained after cope rearrangement to geijerene.
(DOCX)

Materials and Methods S1 (DOCX)
