# Supplementary figures and images for: Subterranean, Herbivore-Induced Plant Volatile Increases Biological Control Activity of Multiple Beneficial Nematode Species in Distinct Habitats
Source: PLoS One. 2012 Jun 27;7(6):e38146. doi: 10.1371/journal.pone.0038146 (PMC3384653; doi:10.1371/journal.pone.0038146)

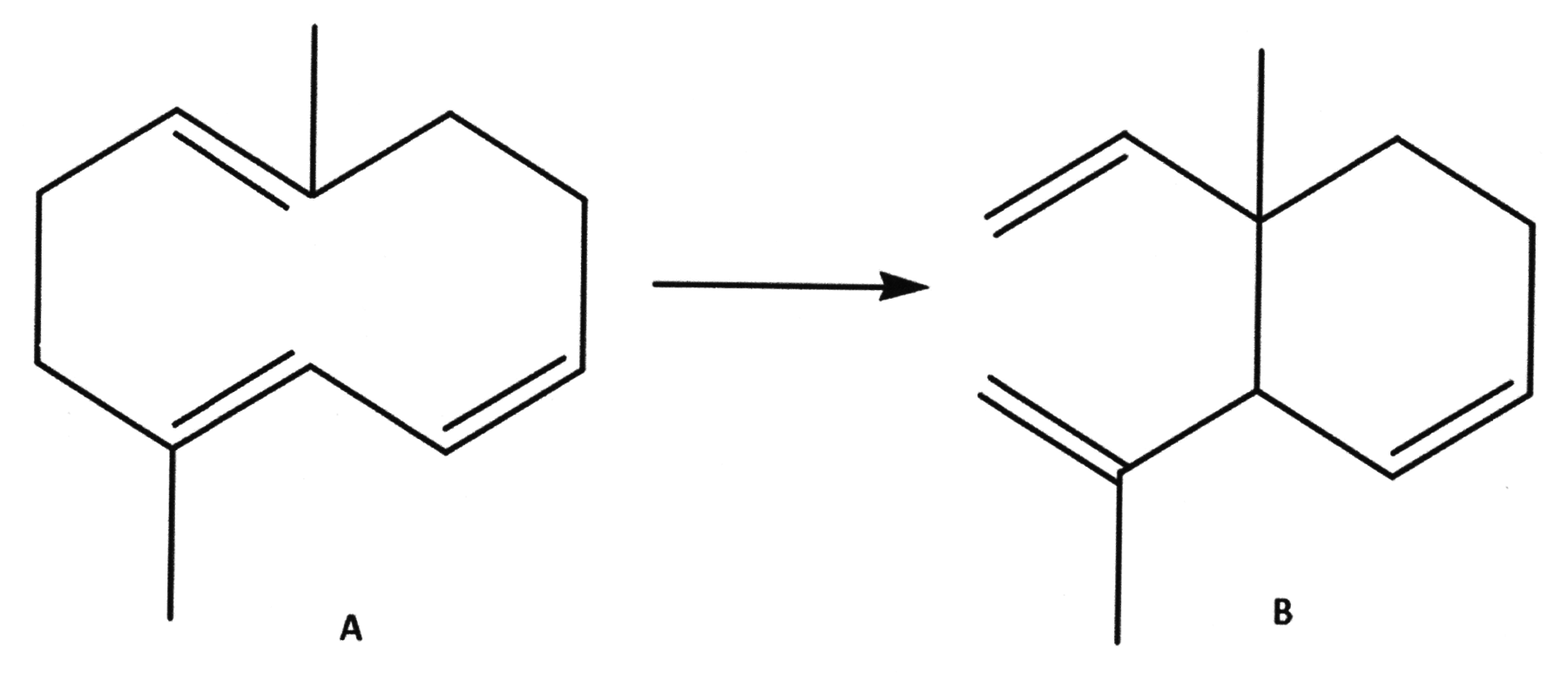

Supplement: Figure S1 — Conversion of pregeijerene (A) to geijerene (B). (TIFF) [file pone.0038146.s001.tif]

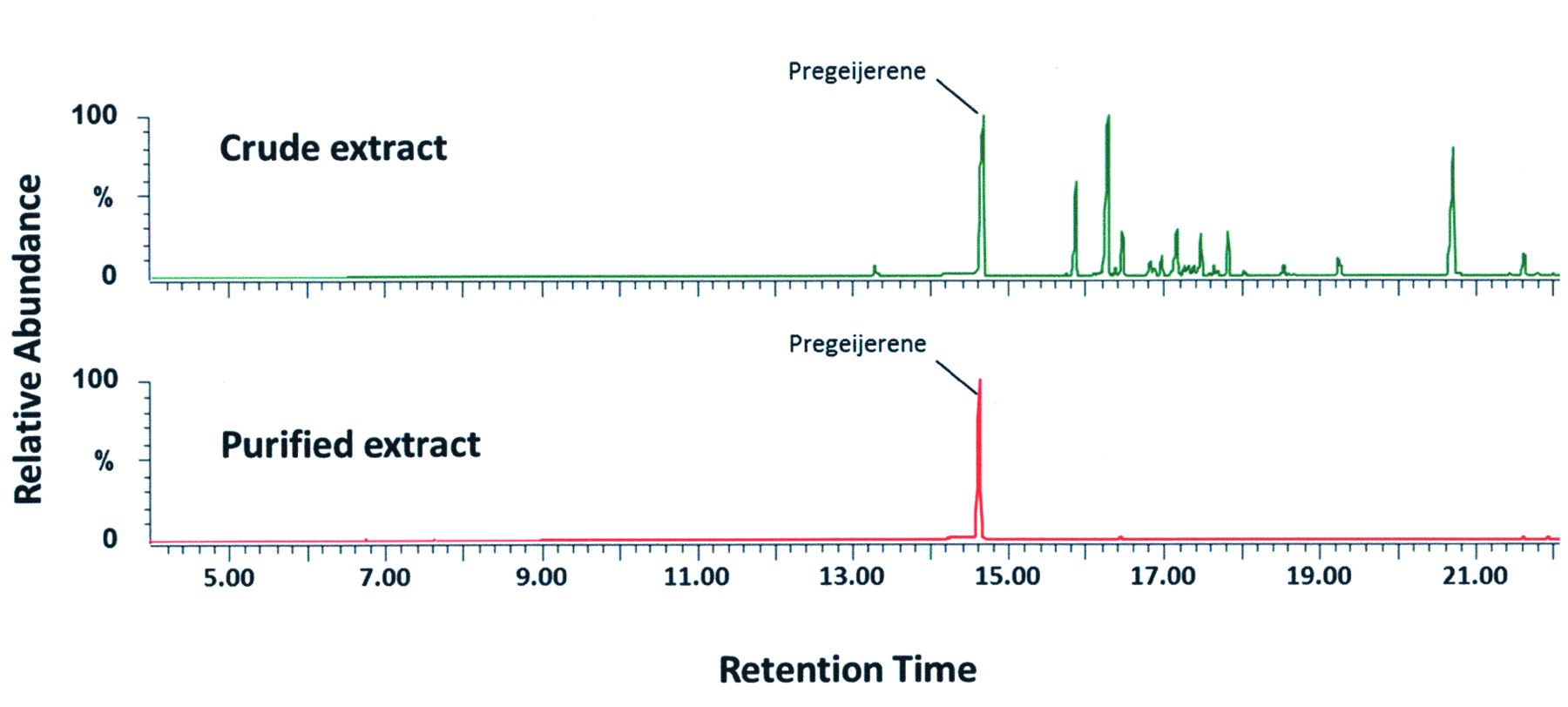

Supplement: Figure S2 — Chromatograms showing the initial crude extract prior to purification and final purified pregeijerene. The Y-axis represents relative abundance or the ratio of a mass peak profile area to that of another. These were calculated as ratios of the sums of areas used to plot the profiles ×100%. (TIFF) [file pone.0038146.s002.tif]

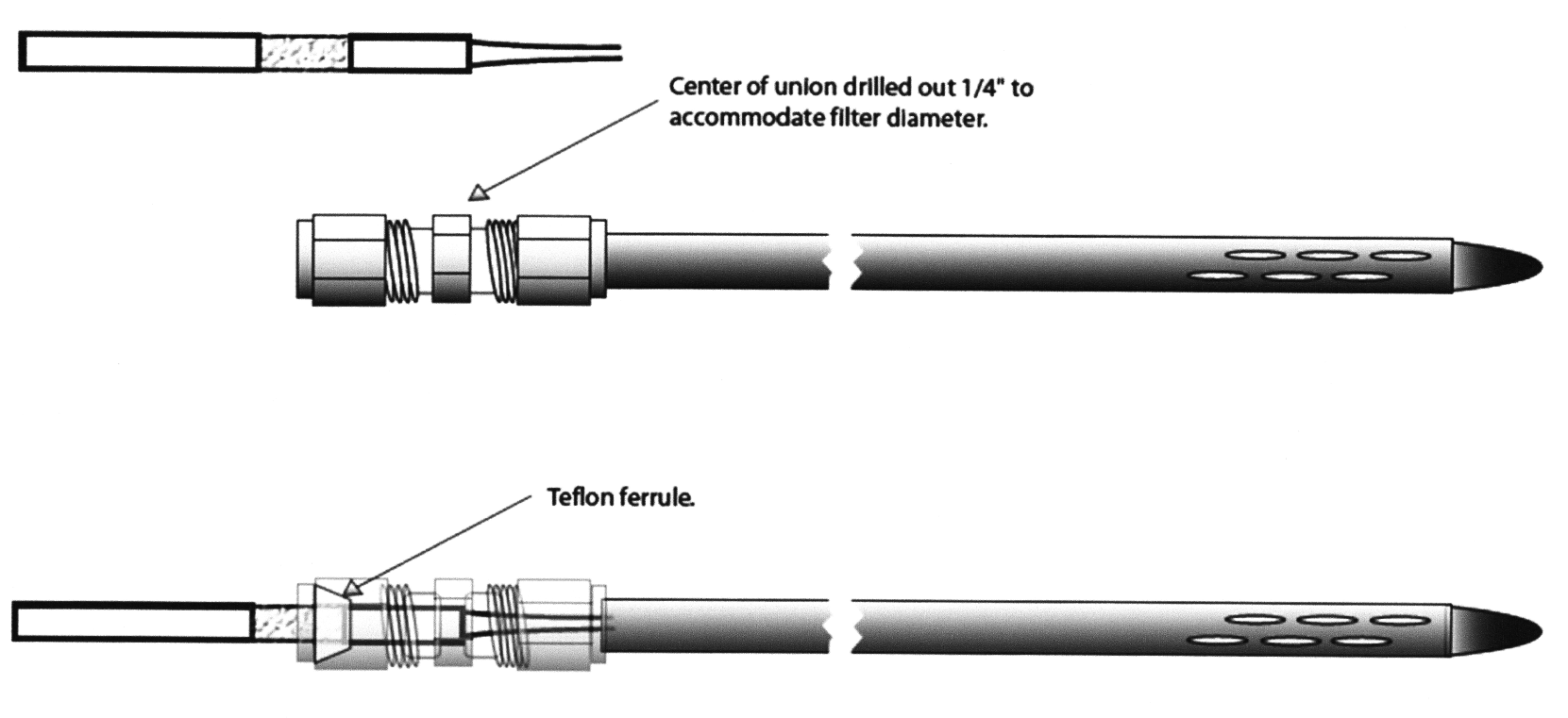

Supplement: Figure S3 — Soil probe design used to sample volatiles belowground. Probe is inserted into soil and connected to a vacuum pump. (TIFF) [file pone.0038146.s003.tif]

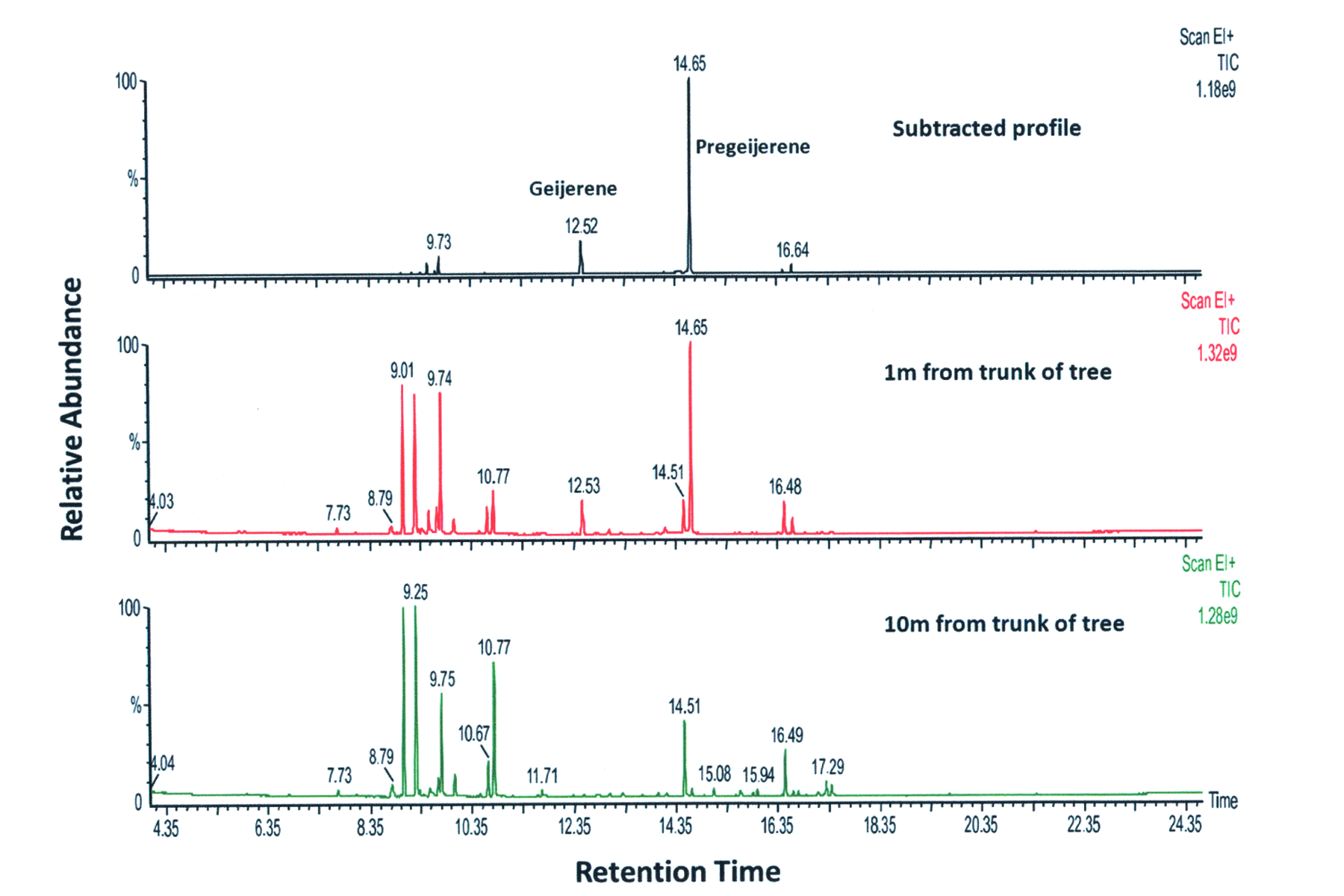

Supplement: Figure S4 — Chromatograms of volatiles taken from intact citrus roots in the field at one and 10 m distances from the trunk of the tree. The Y-axis represents relative abundance or the ratio of a mass peak profile area to that of another. These were calculated as ratios of the sums of areas used to plot the profiles ×100%. (TIFF) [file pone.0038146.s004.tif]

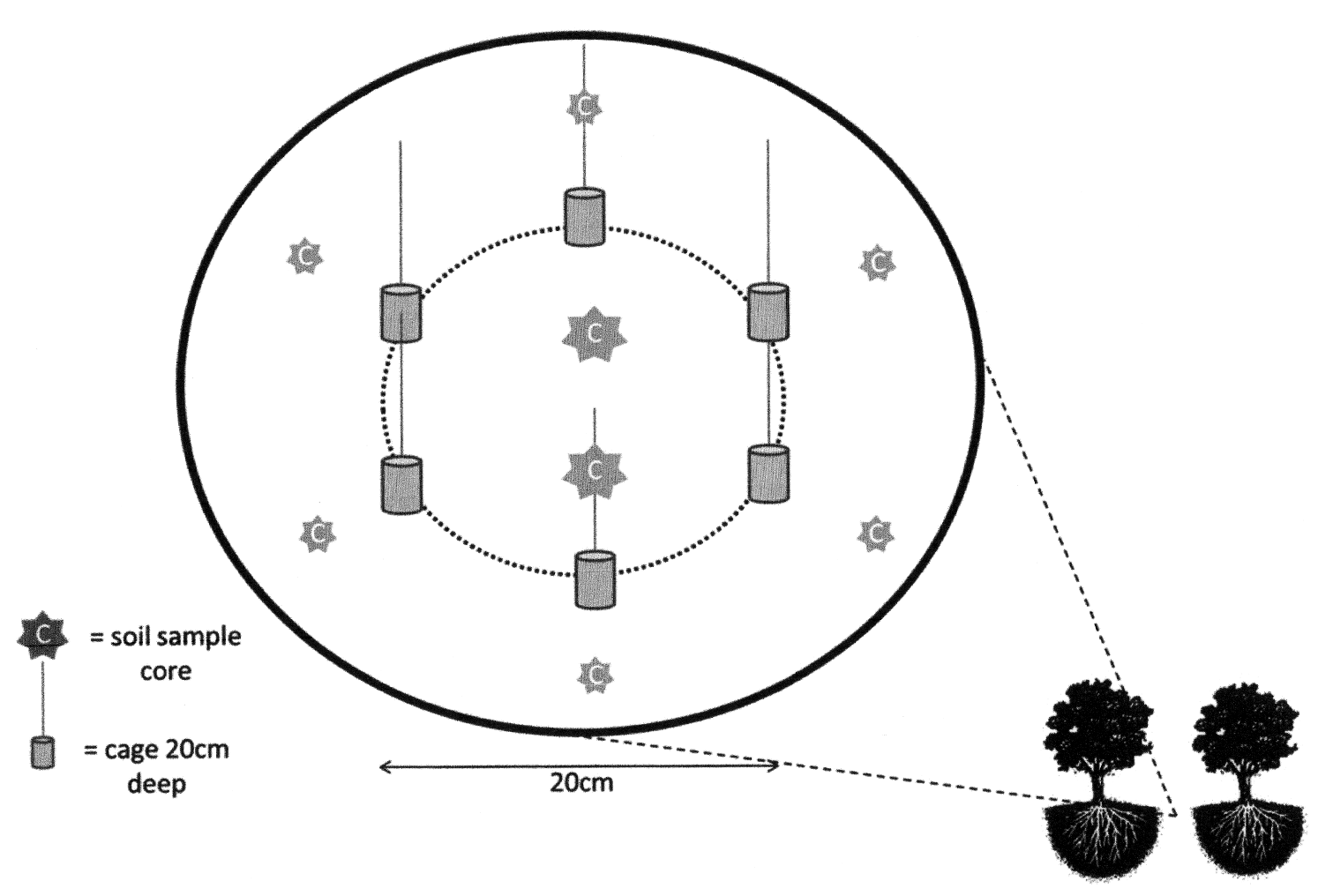

Supplement: Figure S5 — Schematic diagram of the deployment and sampling procedure for field experiments in which sentinel traps with root weevils were deployed with or without HIPVs. One treatment replicate is depicted. (TIFF) [file pone.0038146.s005.tif]
