# Supplementary material for: Subterranean, Herbivore-Induced Plant Volatile Increases Biological Control Activity of Multiple Beneficial Nematode Species in Distinct Habitats
Source: PLoS One. 2012 Jun 27;7(6):e38146. doi: 10.1371/journal.pone.0038146 (PMC3384653; doi:10.1371/journal.pone.0038146)

S Table 1. ^1^H (600 MHz), ^13^C (151 MHz), HMBC and NOESY NMR spectroscopic data for pregeijerene in C_6_D_6_.^13^C was also detected directly (126 MHz) using a 5 mm Cryoprobe. Chemical shifts referenced to residual proton signal in C_6_D_6_ benzene δ(^1^H) = 7.16 ppm for ^1^H and δ(C_6_D_6_H) = 128.2 ppm for ^13^C.

| Position | δ ^13^C [ppm] | δ ^1^H [ppm] | J coupling constants [Hz] | HMBC correlations (C.No) | NOE peaks |
| --- | --- | --- | --- | --- | --- |
| 1 | 140.6 |  |  |  |  |
| 2 | 125.2 | 1H 4.83^#^ | ddt J = 11.5, 4.9, 1.4 |  | 2.45* |
| 3 | 27.6 | 2H 2.06, 1.94 | 2.06, 1H, m  1.94, 1H, m |  | 1.94-1.19* |
| 4 | 39.8 | 2H 2.08, 1.67 | 2.08, 1H, dt J = 11.5, 3.4  1.67, 1H, dt J = 4.4, 12.0 | 1.67-C6, C3 (weak), CH3 of C5 |  |
| 5 | ^$^ |  |  |  |  |
| 6 | 128.9 | 1H 5.39 | br d J = 9.7 | C4, C8 | *2.28, *1.67 |
| 7 | 130.0 | 1H 6.52^#^ | t J = 10 |  | 1.49* |
| 8 | 127.5 | 1H 5.53^#^ | ~dt J = 10.0, 8 |  |  |
| 9 | 29.5 | 2H 2.28, 1.97 | 2.28, 1H, m  1.97, 1H, m |  | 1.97-1.19* |
| 10 | 39.1 | 2H 1.73, 2.45 | 1.73, 1H, dt J = 4.6, 12.8  2.45, 1H, ~ddd J = 12.8, 6.0, 1.9 |  | 1.73-1.19* |
| CH3-C1 | 20.6 | 3H 1.19 | d J = 1.1 | C1, C2, C10 | **1.73, **1.96/1.97 |
| CH3-C5 | 16.2 | 3H 1.49 | s | C6, C4 | **6.52, **2.08, **1.94/1.97 |

* weak NOEs observed with 2D NOESY experiment at 24C, **observed from 1D NOE difference experiments at 10C. ^#^Chemical shifts are temperature sensitive. ^$^We think carbon chemical shifts of C5 and C6 overlap. Carbons numbered based on Jones and Sutherland (1968).


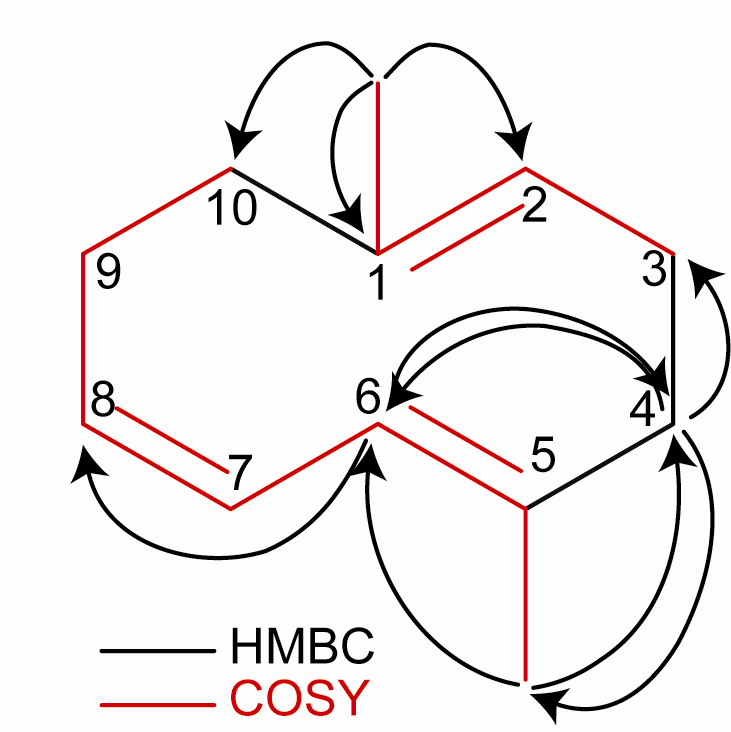

Supplement: Table S1 — 1H (600 MHz), 13C (151 MHz), HMBC and NOESY NMR spectroscopic data for pregeijerene in C6D6.13C was also detected directly (126 MHz) using a 5 mm Cryoprobe. Chemical shifts referenced to residual proton signal in C6D6 benzene δ(1H) = 7.16 ppm for 1H and δ(C6D6H) = 128.2 ppm for 13C. (DOCX) [file pone.0038146.s006.docx]
