# Supplementary material for: Subterranean, Herbivore-Induced Plant Volatile Increases Biological Control Activity of Multiple Beneficial Nematode Species in Distinct Habitats
Source: PLoS One. 2012 Jun 27;7(6):e38146. doi: 10.1371/journal.pone.0038146 (PMC3384653; doi:10.1371/journal.pone.0038146)
Supplement: Table S2 — 1H (600 MHz), 13C (151 MHz), HMBC and NOESY NMR spectroscopic data for geijerene in C6D6.13C was also detected directly (126 MHz) using a 5 mm Cryoprobe. Chemical shifts referenced to residual proton signal in C6D6 benzene δ(1H) = 7.16 ppm for 1H and δ(C6D6H) = 128.2 ppm for 13C. For convenience, the pregeijerene numbering is retained after cope rearrangement to geijerene. (DOCX) [file pone.0038146.s007.docx]

S Table 2. ^1^H (600 MHz), ^13^C (151 MHz), HMBC and NOESY NMR spectroscopic data for geijerene in C_6_D_6_.^13^C was also detected directly (126 MHz) using a 5 mm Cryoprobe. Chemical shifts referenced to residual proton signal in C_6_D_6_ benzene δ(^1^H) = 7.16 ppm for ^1^H and δ(C_6_D_6_H) = 128.2 ppm for ^13^C. For convenience, the pregeijerene numbering is retained after cope rearrangement to geijerene.

| Position | δ ^13^C [ppm] | δ ^1^H [ppm] | J coupling constants [Hz] | HMBC correlations (C.No) | Unique NOESY peaks |
| --- | --- | --- | --- | --- | --- |
| 1 | 38.0 |  |  |  |  |
| 2 | 149.0 | 1H 5.86 | dd J = 17.5, 10.8 |  |  |
| 3 | 110.6 | 2H 4.99, 4.94 | 4.99, 1H, dd J=17.5, 1.3  4.94, 1H, dd J = 10.8, 1.3 | 4.99 - C1  4.95 - C1 | 4.99 - 0.96 |
| 4 | 114.2 | 2H 4.82, 4.97 | 4.82, 1H, br s  4.97, 1H, m |  |  |
| 5 | 146.7 |  |  |  |  |
| 6 | 51.5 | 1H 2.7 | quintet J = 2.7 |  |  |
| 7 | 126.2 | 1H 5.66 | dddd J = 10.1, 2.2, 3.5, 3.5 |  |  |
| 8 | 129.9 | 1H 5.59 | dddd J = 10.1, 3.2, 2.1, 2.1 |  |  |
| 9 | 22.6 | 2H 1.91 | m |  | 0.96 |
| 10 | 33.4 | 2H 1.43 | m |  |  |
| CH3-C1 | 20.9 | 3H 0.96 | s | C1, C2, C6, C10 | 1.72 |
| CH3-C5 | 24.3 | 3H 1.72 | br s | C5, C4, C6 | 0.96 |

**
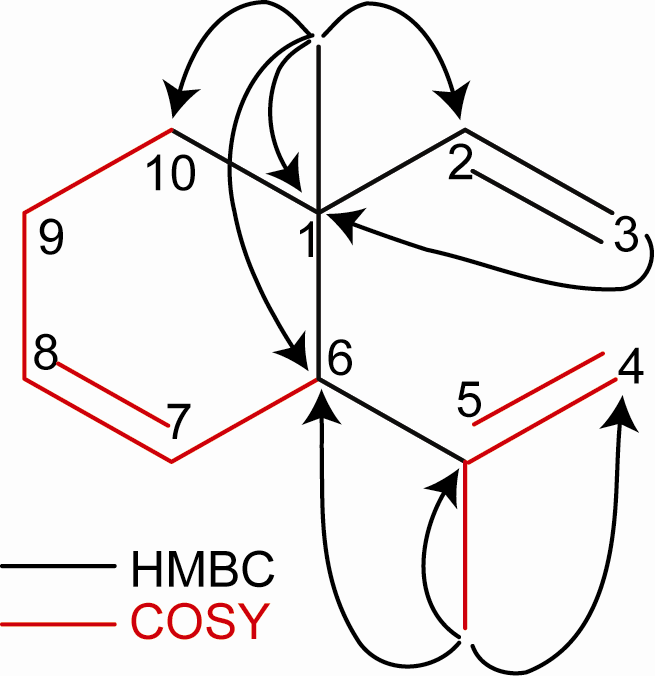
**
