# Supplementary material for: Subterranean, Herbivore-Induced Plant Volatile Increases Biological Control Activity of Multiple Beneficial Nematode Species in Distinct Habitats
Source: PLoS One. 2012 Jun 27;7(6):e38146. doi: 10.1371/journal.pone.0038146 (PMC3384653; doi:10.1371/journal.pone.0038146)
Supplement: Materials and Methods S1 — (DOCX) [file pone.0038146.s008.docx]

**Supplemental Material and Methods**

GC-MS Analysis

All samples were injected as one μl aliquots of dichloromethane extract onto a gas chromatograph (HP 6890) equipped with 30 m×0.25-mm-ID, 0.25 μm film thickness DB-1 or DB35 capillary column (Agilent, Palo Alto, CA, U.S.A.), interfaced to a 5973 or 5975 Mass Selective Detector (Agilent), in both electron impact (EI) and chemical ionization (CI) modes. Samples were introduced using either splitless injection at 220°C or by cold on column injection. In the second case, a one m fused silica deactivated retention gap was added between injector and analytical column and the injector was programmed to follow the oven temperature. The column was held at 35°C for one min after injection and then programmed to change at 10°C/min to 260°C. The carrier gas used was helium at an average flow velocity of 30 cm/s. Isobutane was used as the reagent gas for chemical ionization, and the ion source temperature was set at 250°C in CI and 220°C in EI. EI Spectra library search was performed using a floral scent database compiled at the Department of Chemical Ecology, Göteborg Sweden, the Adams2 terpenoid/natural product library (Allured Corporation, 72) and the NIST05 library. When available, mass spectra and retention times were compared to those of authentic standards in addition to internal standard [nonyl-acetate (4µg/µl)].

Isolation and Purification of Pregeijerene

Although pregeijerene (1,5-dimethylcyclodeca-1,5,7-triene) was collected from citrus roots damaged by *D. abbreviatus*, it was necessary to find an alternative source richer in the pure compound for laboratory bioassay and field testing. Hydrodistilled common rue (*Ruta graveolens*) essential oil contains geijerene as a major constituent (67% of the total volatile compounds) [1]. However, at temperatures exceeding 120ºC [Fig. S1], the macrocyclic pregeijerene will rearrange to geijerene; thus, by on-column analyses of common rue root extracts we found, as anticipated [2], large quantities of pregeijerene rather than geijerene. For isolation of pregeijerene, rue roots were crushed in dichloromethane. GC-MS analyses revealed that pregeijerene constituted approximately 95% of the terpene content, in addition to large quantities of more polar compounds, mostly furanocoumarins. To remove the furanocoumarins, the dichloromethane extract was first eliminated by gently evaporating the sample to a small volume (0.5ml) and was re-suspended in 4 ml of pentane. After centrifugation, the supernatant was again gently concentrated and re-suspended in 4 ml of pentane and again centrifuged to remove solids. An attempt to use a silica column resulted in a partial conversion of pregeijerene to co-geijerene. The yellow solution was therefore slowly passed through a diol column, successfully removing the cyanocoumarins while maintaining intact pregeijerene (Fig. S2). The two remaining impurities were removed by first repeatedly partitioning the hexane extract with methanol followed by a slow filtering through a quartenaryamin ion exchange column. The final hexane solution was analyzed by GC-MS for purity and by GC-FID with nonyl acetate as an internal standard for quantification (Fig S2). Serial dilutions were made from this extract providing five concentrations of pregeijerene (8.0, 0.80, 0.08, 0.008, and 0.0008 µg/µl).

NMR analysis of Pregeijerene

Pregeijerene was purified for nuclear magnetic resonance (NMR) using preparative GC, as a mixture of pregeijere and geijerene 70:30 ratio. The pregeijerene and geijerene mixture (~60 ug) in ~150 µL of C_6_D_6_ (Cambridge Isotope Laboratories Inc.) was placed in a 2.5 mm NMR tube (Norell). One-dimentional ^1^H and nuclear overhauser enhancement (NOE) difference experiments and two-dimensional NMR spectroscopy, including gradient correlation spectroscopy, heteronuclear single-quantum coherence, heteronuclear multiple-bond correlation and NOE spectroscopy were used to characterize pregeijerene. All 2D NMR spectra were acquired at 24°C and an additional 1D NOE difference experiment was conducted at 10°C using a 5-mm TXI CryoProbe and a Bruker Avance II 600 console (600 MHz for ^1^H, 151 MHz for ^13^C). Residual C_6_D_6_ was used to reference chemical shifts to δ(C_6_H_6_) = 7.16 ppm for ^1^H and δ(C_6_H_6_) = 128.2 ppm for ^13^C [3]. NMR spectra were processed using Bruker Topspin 2.1 and MestreLabsMestReNova software packages. Numbering is based on Jones and Southerland [4]. The H and ^13^C NMR data in C_6_D_6_ are presented for pregeijerene and geijerene in Tables S1 and S2 because the original NMR data was obtained in carbon tetrachloride solution.

The ^1^H NMR data (Table S1) for pregeijerene with reported proton chemical shifts and J-couplings for pregeijerene A [4] are consistent, but not with pregeijerene B (Cool & Adams 2003#42). Jones and Southerland [4] did not report ^13^C NMR data, thus we compared the ^13^C NMR data with Germacrene C containing a cyclodecadiene ring like pregeijerene with the exception of an isopropyl substitution at C8 position. Both ^1^H and ^13^C NMR data agreed with germacrene C [5] except for carbons adjacent to C8 as expected. The two-dimentional NOESY experiment at room temperature (24°C) resulted in two very weak NOE. The flexible cyclodecadiene ring was found to exist in three different conformational isomers for germecrine A at or lower than 25°C [6]. Therefore, NOE difference experiments were conducted on the two methyl groups at C1 and C5 at 10°C, above freezing temperature, and 30°C in C_6_D_6_. Overall NOEs were small, but signal intensity was better at 10°C for NOE difference experiments. The protons of methyl group at C5 had NOEs to proton 6.52 of C7, 2.08 of C4 and 1.94 of C3/1.97 of C9. The protons of the methyl group at C1 had NOEs to 1.73 of C10 and 1.97 of C9/.94 of C3. The NOE results agree with pregeijerene and flexible cyclodecadiene ring structures [4-6]. In addition, we found that chemical shifts of protons at C2, C7 and C8 are sensitive to temperature changes.

**References**

1. Kuzovkina I, Szarka S Héthelyi, É (2009) Composition of essential oil in genetically transformed roots of *Ruta graveolens*. Russ J Plant Physl 56:846-851.

2. Kubeczka K, Ullmann I (1980) Occurrence of 1, 5-dimethylcyclodeca-1, 5, 7-triene (pregeijerene) in *Pimpinella* species and chemosystematic implications. Biochem Syst Ecol 8: 39-41.

3. Fulmer GR, Miller AJM, Sherden NH, Gottlieb HE, Nudelman A, et al. (2010) NMR chemical shifts of trace impurities: common laboratory solvents, organics, and gases in deuterated solvents relevant to the organometallic chemist. Organometallics 29: 2176–2179.

4. Jones RVJ, Sutherland, MD (1968) 1,5-Dimethylcyclodeca-1,5,7-Triene, the precursor of geijerene in *Geijera Parviflora* (Lindley). Aust J Chem 21: 2255–2264.

5. Colby SM, Crock J, Dowdle-Rizzo B, Lemaux PG, Croteau R. (1998) Germacrene C synthase from *Lycopersicon esculentum* cv. VFNT cherry tomato: cDNA isolation, characterization, and bacterial expression of the multiple product sesquiterpene cyclase. Proc Natl Acad Sci USA 95: 2216–2221.

6. Faraldos JA, Wu S, Chappell J, Coates RM (2007) Conformational analysis of (+)-germacrene A by variable temperature NMR and NOE spectroscopy. Tetrahedron 63: 7733–7742.
